# Frequency and Risk Factors of Cyclosporine-Induced Neurotoxicity in Allogeneic Stem Cell Transplant Recipients

**DOI:** 10.7759/cureus.19824

**Published:** 2021-11-22

**Authors:** Asma Danish, Sarah I Mughal, Uzma Zaidi, Shabnam Dildar, Shafaq Samad, Aisha Jamal, Zainab Sharif, Tahir Shamsi

**Affiliations:** 1 Department of Clinical Hematology, National Institute of Blood Disease and Bone Marrow Transplantation, Karachi, PAK; 2 Department of Research and Development, National Institute of Blood Disease and Bone Marrow Transplantation, Karachi, PAK; 3 Department of Bone Marrow Transplantation, National Institute of Blood Disease and Bone Marrow Transplantation, Karachi, PAK; 4 Chemical Pathology, National Institute of Blood Disease and Bone Marrow Transplantation, Karachi, PAK; 5 Hematology, National Institute of Blood Disease and Bone Marrow Transplantation, Karachi, PAK

**Keywords:** neurological complications, posterior reversible encephalopathy syndrome (pres), stem-cell transplant, cyclosporine induced neurotoxicity (cin), cyclosporine a

## Abstract

Background and objective

The calcineurin inhibitor cyclosporine A is routinely used for prophylaxis against graft-versus-host-disease (GvHD) in human leukocyte antigen (HLA)-matched allogeneic stem-cell transplant patients and is a major etiological factor for neuropathological symptoms that are reversible in most cases. In this study, we aimed to determine the frequency and risk factors of cyclosporine-induced neurotoxicity (CIN) in HLA-matched allogeneic stem cell transplant patients.

Methods

The study spanned the period from January 2016 to December 2019. Consecutive HLA-matched allogeneic stem-cell transplant patients of all ages were included in the study. Descriptive and risk factor analyses for the development of CIN with respect to age, sex, primary diagnosis, conditioning regimen, electrolyte abnormalities, and cyclosporine trough levels during the neurological episode were performed.

Results

A total of 106 HLA-matched patients with a median age of 6.3 years [interquartile range (IQR): 0.5-46 years], of which 37 (35%) were females, were included in the study. The mean cyclosporine trough level was 500 ±286 mg/dl. Neurological symptoms were found in 27 (26%) patients. A total of 14 (13%) patients were diagnosed with CIN. The frequency of other neurological symptoms included headache in 46 (43%), disorientation in 17 (16%), seizures in 12 (11%), visual disturbance in 11 (10%), and aphasia in seven (7%) patients. Posterior reversible encephalopathy syndrome (PRES) was found in six (6%) patients. All patients with CIN had hypertension and none had a fever. Multivariate logistic analysis showed that the presence of seizures [odds ratio (OR): 10.0, p<0.001] and the absence of fever (OR: 0.02, p<0.001) were associated with the diagnosis of CIN.

Conclusion

The prevalence of CIN is not uncommon (13%) in patients receiving cyclosporine for GvHD prophylaxis. Neurological complications, especially seizures, are common in CIN, and fever might indicate an alternative diagnosis. Prompt recognition of neurological signs and symptoms and early intervention can halt the progression of the disease.

## Introduction

Neurological complications are the predominant cause of transplant-related morbidity and mortality in hematopoietic stem cell recipients [1]. A wide range of neurological symptoms such as seizures, disorientation, confusion, cortical blindness, aphasia, pyramidal and extrapyramidal motor weakness, ataxia, and hallucinations are common both in early and in late phases after transplantation [2]. A multitude of causative factors has been identified, including cerebrovascular bleeding, metabolic disturbances (hypocalcemia, hypomagnesemia, hypoglycemia), opportunistic bacterial, fungal, viral, and protozoal infections, and drug toxicity, particularly related to cyclosporine A [3]. Among other causes of neuropathological symptoms, the calcineurin inhibitor cyclosporine A, which is routinely used for graft-versus-host-disease (GvHD) prophylaxis in human leukocyte antigen (HLA)-matched allogeneic stem cell transplant patients, is a major etiological factor but reversible in most cases after the withdrawal of the drug. While its common side effects include renal and hepatotoxicity, gingival hyperplasia, hypertrichosis, hypertension, and tremors, its neurological side effects are also pronounced and reported in 4-11% of patients undergoing transplants [4]. Although it is rarely a cause of mortality, its necessitated withdrawal as a major anti-GvHD drug leads to serious implications on clinical outcomes of transplants [5]. While assessing the risk factors for the development of cyclosporine A toxicity, metabolic derangements like hypomagnesemia, hypo/hypercalcemia, hyponatremia, and pre-existing neurological disturbances appear to play a significant part. Cyclosporine levels apparently have no effect on its development as neurotoxicity has been observed at both therapeutic and toxic levels [6].

Cyclosporine is also a major risk factor for the development of posterior reversible encephalopathy syndrome (PRES), which is a neurological syndrome with specific clinical symptoms and distinctive MRI findings. Although there are varied etiological factors for its development, in transplant settings, cyclosporine A is a principal contributor [7], and it typically presents with seizures, acute encephalopathy syndrome, and visual symptoms, while dysarthria, incoordination of the limbs, paresis, sensory deficits, and visual hallucinations are atypical features. Its characteristic neuroimaging features a hyperintense signal, distributed in the parietal and occipital lobes on FLAIR images [8]. Although a significant cause of morbidity, the prognosis of cyclosporine A neurotoxicity is generally good, and posterior leukoencephalopathy usually resolves completely with dose reduction or drug withdrawal [9].

The objective of this study was to determine the frequency and risk factors of neurotoxicity related to cyclosporine A in full-matched allogeneic stem cell transplant recipients in a bone marrow transplant setting at an under-resourced facility.

## Materials and methods

This was a retrospective study conducted at the Department of Clinical Hematology in collaboration with the Department of Bone Marrow Transplant at the National Institute of Blood Disease and Bone Marrow Transplantation (NIBD), Karachi, Pakistan from January 2016 to December 2019. All pediatric and adult patients who underwent HLA-matched allogeneic stem-cell transplantation were identified from the electronic database system. All variables including follow-up information of 100 days post-transplantation were collected for these patients by the primary author via a manual chart review on a structured data collection tool. All data were retrospectively collected, and patient identification information was removed to ensure anonymity. Institutional review board (IRB) approval from the NIBD Ethical Committee was obtained prior to the start of this study. The IRB approval number was NIBD/RD-225/12-2020.

Study variables

Variables that were collected included demographics, pre-transplant diagnosis, date of transplant, transplant procedures, and post-transplantation neurological symptoms including headache, vomiting, tremors, seizures, disorientation, visual symptoms, cyclosporine trough levels, as well as magnesium, calcium, sodium, potassium, and glucose levels at the time of the neurological event. CT scan or MRI findings from the post-transplantation period were also collected.

Definitions for the diagnosis of cyclosporine-induced neurotoxicity (CIN) and posterior reversible encephalopathy syndrome (PRES)

The diagnosis of CIN was considered when a bone marrow transplant recipient on cyclosporine A for GvHD prophylaxis developed clinical symptoms of headache, vomiting, hypertension, tremors, seizures, aphasia, cortical blindness, or disorientation [10], and had specific electrolyte imbalances and outcomes assessed after the discontinuation of cyclosporine had shown improvement. CIN diagnosis was confirmed if the presence of the above-mentioned clinical signs and symptoms correlated with CT/MRI scan or revealed PRES. Diagnosis of PRES was based on characteristic MRI findings and compatible clinical symptoms. No CIN was labeled when patients having neurologic signs and symptoms had non-specific electrolyte imbalances, if the symptoms remained the same or worsened on cyclosporine withdrawal, and were not radiologically proven. Patients having a prior central nervous system (CNS) pathology were not considered to have CIN.

Chemotherapeutic regimens

Based on the hematopoietic stem-cell source, donor type, patient age, comorbidities, and underlying disease, conditioning regimens were administered according to institutional protocols. Conditioning regimen intensity was classified as myeloablative using cyclophosphamide, busulphan, and anti-thymocyte globulin (ATG), or non-myeloablative using fludarabine and ATG or reduced-intensity conditioning (RIC) [11]. Levetiracetam prophylaxis (10 mg/kg dose) was given to all patients receiving busulfan as a part of the conditioning regimen. GvHD prophylaxis in allogeneic hematopoietic stem cell transplant for beta-thalassemia major (BTM), aplastic anemia, and acute leukemia received IV cyclosporine (3.5 mg/kg/day from day two) with weekly monitoring of cyclosporine trough levels, combined with IV methotrexate (15 mg/m^2^ on day one followed by 10 mg/m^2^ on days three, six, and 11).

Data analysis

Descriptive analysis with means and two standard deviations (SD) and medians with interquartile range (IQR) were used to report continuous variables when found appropriate. Frequencies with proportions documented as percentages were reported for categorical variables. Student’s t-test as the test of significance for comparing continuous with categorical variables and cross-tabulation with chi-square test of significance for categorical variables were performed. Univariate and multivariable analysis was performed for CIN association with risk factors, pretransplant diagnoses, neurological symptoms (except PRES since it was used to diagnose CIN), and laboratory abnormalities. A p-value of <0.05 was considered statistically significant. Data were analyzed using SPSS Statistics version 21.0 (IBM, Armonk, NY).

## Results

Of the 106 patients included in the study, 37 (35%) were females. The median age was 6.3 years [interquartile range (IQR): 0.5-46 years]. There were 44 (41.5%) patients who were ABO-mismatched. The most common diagnosis in patients were BTM (n=55, 52%), aplastic anemia (AA) (n=31, 29%), and acute myeloid leukemia (n=6, 6%) (Table 1). In terms of conditioning regimens, there were 68 (64%) patients who received a myeloablative regimen, while 34 (32%) received a non-myeloablative regimen, and four (4%) received a reduced-intensity regimen.

**Table 1:** Comparison of characteristics between patients with and without calcineurin inhibitor-induced neurotoxicity (CIN) CIN: calcineurin inhibitor-induced neurotoxicity; PRES: posterior reversible leukoencephalopathy syndrome; SD: standard deviation

Variables		No CIN (n=92)	CIN (n=14)	P-value
Median age (IQR), years		6.0 (0.5-46)	9.5 (1.4-38)	0.529
Females, n (%)		32 (35)	5 (36)	1.0
ABO mismatch, n (%)		36 (39)	8 (47)	0.526
Mean cyclosporine level ±SD (mg/dl)		485.7 ±247.5	613.4 ±394.7	0.139
Conditioning regimen, n (%)	Myeloablative	58 (63)	10 (71)	0.669
	Non-myeloablative	30 (33)	4 (29)	
	Reduced-intensity	4 (4)	0	
Hypertension, n (%)		38 (41)	14 (100)	<0.001
Headaches, n (%)		33 (36)	13 (93)	<0.001
Visual disturbance, n (%)		3 (3)	8 (57)	<0.001
Tremors, n (%)		2 (2)	4 (29)	0.003
Seizures, n (%)		3 (3)	9 (64)	<0.001
Aphasia, n (%)		2 (2)	5 (36)	<0.001
Disorientation, n (%)		8 (9)	9 (64)	<0.001
Fever, n (%)		40 (44)	0	0.001
PRES, n (%)		0	6 (43)	<0.001
Mean sodium level ±SD (mg/dl)		139.2 ±4.1	138.9 ±3.4	0.846
Mean potassium level ±SD (mg/dl)		4.0 ±0.6	3.9 ±0.6	0.526
Mean calcium level ±SD (mg/dl)		9.0 ±0.6	8.9 ±0.9	0.429
Mean magnesium level ±SD (mmol/dl)		1.8 ±0.3	1.4 ±0.1	<0.001
Mean glucose level ±SD (mg/dl)		164.5 ±45.1	159.2 ±49.9	0.958
Mean urea level ±SD (mg/dl)		18.6 ±4.1	17.6 ±3.7	0.402
Mean creatinine level ±SD (mg/dl)		0.7 ±0.3	0.8 ±0.5	0.617

Overall, the mean cyclosporine trough level post-transplant was 500 ±286 mg/dL. Neurological symptoms were found in 27 (26%) patients. There were 14 (13%) patients with confirmed or suspected CIN, of which confirmed CIN was found in six (6%) patients while eight (7.5%) patients were suspected (not confirmed radiologically but the symptoms improved after cyclosporine withdrawal) to have CIN. The remaining 13 patients with neurological symptoms either had no findings on CT imaging or did not improve with cyclosporine withdrawal and were labeled as not having CIN.

Characteristics of cyclosporine-induced neurotoxicity (CIN) patients

When comparing the patients with and without CIN, age, gender, distribution of diagnosis, conditioning regimen, and cyclosporine levels were not significantly different between the two groups. However, the presence of neurological symptoms was significantly more in patients with CIN. All patients with CIN had hypertension and none had a fever. The mean sodium, potassium, calcium, creatinine, glucose, and urea levels were not different between the two groups; however, a lower level of magnesium was seen in patients with CIN (Table 2).

**Table 2:** The distribution of patients according to their diagnosis CIN: calcineurin inhibitor-induced neurotoxicity; BTM: beta-thalassemia major; AML: acute myelocytic leukemia; SCID: severe combined immunodeficiency; FA: Fanconi anemia; ALL: acute lymphoblastic leukemia; PNH: paroxysmal nocturnal hemoglobinuria; CML: chronic myeloid leukemia

Primary diagnosis	No CIN (n=92)	CIN (n=14)	P-value
BTM, n (%)	48 (52.5)	7 (50)	0.711
Aplastic anemia, n (%)	27 (29.5)	4 (29)	
AML, n (%)	5 (5)	1 (7)	
SCID, n (%)	3 (3)	0	
FA, n (%)	3 (3)	0	
ALL, n (%)	2 (2)	0	
Gaucher disease, n (%)	1 (1.25)	0	
PNH, n (%)	1 (1.25)	0	
Sideroblastic anemia, n (%)	1 (1.25)	0	
Agammaglobulinemia, n (%)	1 (1.25)	0	
CML, n (%)	0	1 (7)	
Hurler, n (%)	0	1 (7)	

On univariate analysis, the clinical findings of hypertension, fever, aphasia, seizures, disorientation, visual disturbance, headache, and magnesium level were found to be significant. When included in a multivariate logistic regression model, the absence of fever and the presence of seizures were found to be associated with the diagnosis of CIN (Table 3).

**Table 3:** Multivariate model for clinical findings associated with calcineurin inhibitor-induced neurotoxicity (CIN)

Variables	P-value	Odds ratio	95% confidence interval	
			Lower	Upper
Magnesium <1.7 mg/dl	0.478	2.0	0.3	1.25
Seizures	<0.001	10.0	0.0	10.0
Headache	0.740	1.5	0.1	1.7
Visual disturbance	0.071	12.5	0.8	100
Disorientation	0.651	0.5	0.02	10
Aphasia	0.5	1.0	0.0	1.0
Fever	0.023	0.02	0.001	0.6
Hypertension	0.114	9.0	0.59	100

## Discussion

Our study showed that CIN is not an uncommon finding in patients who are receiving cyclosporine for prophylaxis of GvHD as a part of their bone marrow transplant. Neurologic complications are a frequent occurrence in allogeneic bone marrow transplant recipients on cyclosporine for GvHD prophylaxis, and second only in frequency after renal complications [12]. The incidence of CIN at our institution was similar to that in the more recently published studies in similar settings [13]. However, the incidence of CIN has been reported to be as high as 25-29% in earlier studies [14]. In our study, we diagnosed some patients with CIN based on clinical signs and symptoms, laboratory parameters, and improvements in the symptoms after the withdrawal of cyclosporine. All patients with confirmed CIN had a diagnosis of PRES based on imaging findings. However, since not all patients underwent CT or MRI scans, the incidence of PRES is likely underreported. The wide-ranging neurological symptoms in patients with CIN, from subtle tremors or mild headaches to encephalopathy and PRES, indicate the need for vigilance with close follow-up for all patients who are on cyclosporine.

PRES is a clinical radiopathologic entity, and it was first described in 1996 as a disease with acute neurological symptoms, which are reversible upon withdrawal of the etiological factor, and specific radiologically proven white matter lesion [15]. The association of PRES with cyclosporine was first described by Adams et al. [16]. The likely pathophysiological process includes the direct toxic effects of cyclosporine on vascular endothelial cells, causing them to release endothelin, prostacyclin, and thromboxane, producing microthrombi and damaging the blood-brain barrier [17].

Previously published studies have shown the association of CIN with numerous factors particularly related to genetics, metabolic and electrolyte disturbances, and transplant procedures [18,19]. Certain studies quote genetic polymorphisms in CYP3A5, and P-glycoprotein encoded by the ABCB1 gene, which may contribute to CIN [20]; however, further studies are required to confirm these postulations.

Certain clinical parameters like arterial hypertension, hypomagnesemia, hyponatremia, hyperkalemia, and hyperglycemia have been shown to have a significant association with CIN. Morgan et al. [21] and Thompson et al. [22] have studied the association of electrolytes with CIN and even suggested that magnesium supplementation might lead to prevention and treatment of neurological symptoms in patients with CIN. In our study, hypomagnesemia was seen in a majority of transplanted patients; however, it was not found to be independently associated with the development of neurotoxicity.

This was a retrospective study with its inherent limitations in the study design. The study setting was an under-resourced environment with patients having no health insurance and all of them paying out of pocket for their care. This often leads to limitations in the number of imaging and laboratory follow-ups that can be performed for these patients. Hence, not all patients were able to obtain a CT or MRI and as a result, some of them were diagnosed with CIN based on the improvement of neurological symptoms after the discontinuation of cyclosporine A. Due to this, the incidence of PRES was likely underreported in this cohort.

## Conclusions

Our study described the findings from a study of full-matched bone marrow transplantation recipients who developed CIN conducted at a single institution. The condition was not uncommon in our patients. Being a reversible entity, early recognition of CIN's vast range of signs and symptoms and prompt intervention along with correction of electrolyte imbalances can reverse and improve the neurological condition.
